# Decomposition of α-Tocopheryl Glycosides in Rat Tissues

**DOI:** 10.1080/15376510802164519

**Published:** 2008-06-23

**Authors:** Małgorzata Knaś, Piotr Wałejko, Jadwiga Maj, Agnieszka Hryniewicka, Stanisław Witkowski, Małgorzata Borzym-Kluczyk, Danuta Dudzik, Krzysztof Zwierz

**Affiliations:** Department of Pharmaceutical Biochemistry, Medical University of Bialystok, Poland; Institute of Chemistry, University of Bialystok, Poland; Department of Pharmaceutical Biochemistry, Medical University of Bialystok, Poland

**Keywords:** Exoglycosidases, HPLC, α-Tocopheryl Glycosides, Vitamin E

## Abstract

**Background:**

The aim of our investigation was to estimate the stability of α-tocopheryl *O*-glycosides in relation to activity of exoglycosidases in selected rat tissues.

**Material and Methods:**

Acetylated glycosides were obtained in glucosidation of α-tocopherol using the Helferich method. The activity of exoglycosidases was determined by the Zwierz et al. method. Protein concentrations were determined by the biuret method. The concentration of released α-tocopherol was determined with the HPLC method.

**Results:**

The comparison of the amount of released α-tocopherol with the amount of released *p*-nitrophenol shows that glycoside bound in **2a–5a** derivatives of α-tocopherol undergoes hydrolysis significantly harder than in appropriate **2b–5b** *p*-nitrophenyl derivatives.

**Conclusion:**

The results indicate that tocopheryl *O*-glycosides are more resistant to enzymatic hydrolysis than appropriate *p*-nitrophenol *O*-glycosides **2a–5a**. Among examined tocopheryl *O*-glycosides, galactoside **4** is the only compound that caused the significant increase in tocopherol concentration, as compared to its endogenic content.

## INTRODUCTION

**FIGURE 1:**
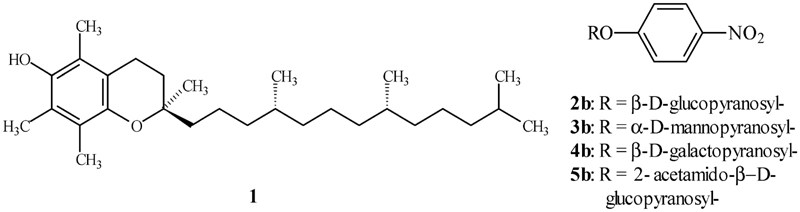
Structure of α-tocopherol (1) and *p*-nitrophenyl glycosides (2b–5b).

Vitamin E (α-tocopherol **1**) (Fig. 1) is regarded as the most important low-molecular lipophilic antioxidant that protects cellular membranes and other phospholipid structures against the destructive activity of oxygen-centered free radicals (Azzi and Stocker 2000; Wang and Quinn 1999). The investigations carried out for decades showed that administration of vitamin E helps in the treatment of such diseases as atherosclerosis and circulatory disorders. It inhibits prostate proliferation and delays progress of Alzheimer and Parkinson diseases and many other disorders connected with disturbances of free radical levels in the organism (Hasty et al. 2007; Negri et al. 1991; Vatassery et al. 1999). However, moderate absorption (10%–70% of content in diet) and long-lasting transport to cells limit the therapeutic use of vitamin E.

One of the possible ways of increasing the bioavailability of vitamin E is to convert free α-tocopherol into *O*-glycoside derivatives. *O*-glycosidic derivatives of vitamin E as prodrugs of higher amphiphilic character show better solubility in body fluids and better permeability across cellular membranes. Thus, *O*-glycosides of α-tocopherol would reach the target place faster and release active molecules of tocopherol as a result of the activity of appropriate exoglycosidases or acidic medium (under ischemic-reperfusion conditions) (Matsuoka et al. 1998; Mizuma et al. 1994, 1992; Veyhl et al. 1998).

**FIGURE 2:**
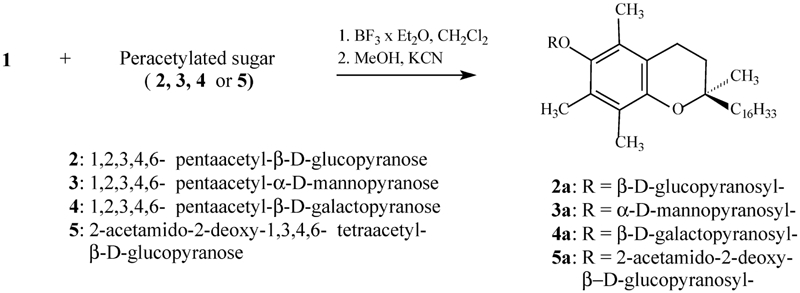
Synthesis of α-tocopheryl *O*-glycosides.

Lahmann and Thiem (1997) have reported that *O*-glycosides of α-tocopherol are resistant toward α- and β-glucosidases isolated from almonds and hen's eggs, respectively. On the other hand, our preliminary studies pointed to the fact that α-tocopheryl galactoside **4a** underwent a slow decomposition in supernatant fluids from homogenates of selected rat tissues (ileum, kidney, etc.) (Witkowski et al. 2004). In another experiment, a prolonged photoprotective effect was observed for α-tocopheryl glucoside (**2a**) during UVB irradiation of naked mice (Niczyporuk et al. 2003). The present paper shows further data concerning the persistence of α-tocopheryl *O*-glycosides (**2a–5a**) (Fig. 2) in the presence of exoglycosidases present in chosen rat tissues (ileum, brain, liver, and kidney).

## MATERIAL AND METHODS

### Synthesis of α-Tocopheryl Glycosides

α-Tocopheryl *O*-glycosides **2a–5a** (Fig. 2) were obtained according to the modified Helferich method (Sch. 1) (Lahmann and Thiem 1997; Witkowski and Walejko 2002). After deacetylation in anhydrous methanol in the presence of KCN, the crude glycosides were purified by column chromatography. The purity of the compounds **2a–5a** was confirmed by ^1^H and ^13^C NMR as well as by HPLC.

### Preparation of tissues

The ileum, brain, liver, and kidney tissues of Wistar rats were homogenized for 90 sec in four volumes of 0.2% of Triton X-100 in 0.15 M KCl. The homogenates were centrifuged at 12,000 × g, for 30 min at 4°C and the supernatants were used for further experiments. Antioxidants–8 mg of 2,6-di-tert-butyl-4-methylphenol (BHT) and 7 mg of ascorbic acid–were added to each sample containing 3.5 mL of the supernatant. Substrates for the activity determination of lysosomal exoglycosidases and Triton X-100 were purchased from Sigma (St. Louis, USA), the solvents for HPLC from Lab-Scan, and the rest of the reagents from the Polish Chemical Reagent Factory (POCh), Gliwice.

### Methods of determination

**FIGURE 3:**
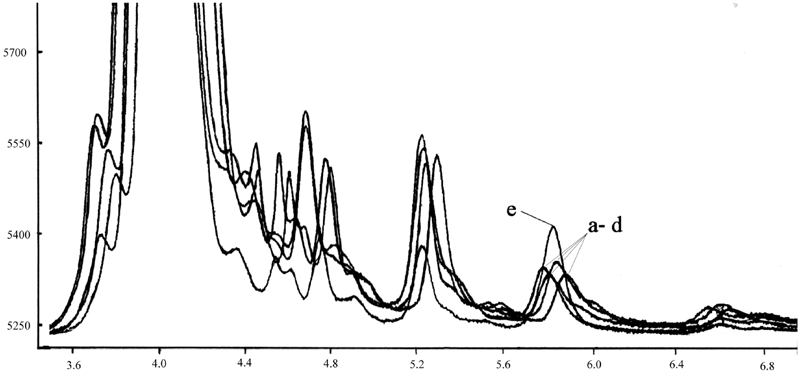
Chromatograms of the extracts from liver tissue (from 50 μL of supernatant): a–d: endogenic α-tocopherol; e: after addition 10 μg of α-tocopherol. 5.7–6.0 free α-tocopherol peak.

In order to determine the activity of exoglycosidases in a supernatant, we used the method of Zwierz et al. (1981). Concentrations of proteins were determined by the biuret method, using serum bovine albumin as a standard (Dawson et al. 1969). The activity of lysosomal exoglycosidases N-acetyl-β-D-hexosaminidase, β-galactosidase, α-mannosidase, and β-glucosidase was determined on the basis of the amount of *p*-nitrophenol (in nmol) released after 60 min incubation at 37°C, of 50 μL of supernatant fluids with *p*-nitrophenyl *O*-glycosides **2b–5b** (Fig. 1). Determination was conducted using a large excess of *p*-nitrophenyl glycosides and therefore the amount of released *p*-nitrophenyl is proportional to the activity of the appropriate enzyme. The release of α-tocopherol from α-tocopheryl *O*-glycosides (α-tocopheryl β-D-glucopyranoside [**2a**], α-tocopheryl β-D-mannopyranoside [**3a**], α-tocopheryl β-D-galactopyranoside [**4a**], α-tocopheryl 2-acetamido-β-D-glucopyranoside [**5a**]) in the presence of exoglycosidases was determined under identical conditions. The action of enzymes was stopped after 2 h of incubation by extraction with hexane (3 × 1 mL). The combined hexane extracts were stored at −10°C. The analyzed samples were evaporated to dryness immediately before the measurement and the residues were dissolved in of hexane (0.5 mL). The amount of released α-tocopherol (**1**) was determined by HPLC. The recovery of method was practically quantitative in the procedure used. The chromatograms obtained from the supernatant before and after addition of the known amount of α-tocopherol (10 μg) are presented in Figure 3.

The HPLC system (LabAlliance) consisted of pumps (III Pump series), the detector UV-VIS (525 Dual-wavelength), and the device for sample introduction (a dosing valve Rheodyne Model 7725i and a 20-μL dosing loop). The column HPLC (25 cm × 4.6 mm, NH_2_-NP, 5 μm) (Supelco) was used. The 3% solution of isopropanol in hexane was a mobile phase. The analysis was conducted at the flow velocity of 1 mL/min, at room temperature, with the detection at 295 nm with the use of Data Ally Chromatography Manager for Windows 95.

The investigation has the approval of a Local Ethical Committee.

## RESULTS

The activity of hepatic exoglycosidases was determined on the basis of the amount of *p*-nitrophenol released after 60 min incubation at 37°C of *p*-nitrophenyl glycosides **2b–5b** in 50 μL of supernatant (Table 1).

**TABLE 1:** Amounts of *p*-nitrophenol (nmol) released after 60 min incubation of *p*-nitrophenyl glycosides 2b–5b in 50 μL of supernatant (n = 8)

Supernatant	2b (nmol) ± SD	3b (nmol) ± SD	4b (nmol) ± SD	5b (nmol) ± SD
Kidney	196 ± 40	10 ± 8	180 ± 20	278 ± 70
Intestine	38 ± 18	6 ± 3	42 ± 5	130 ± 40
Brain	18 ± 7	2.5 ± 0.5	20 ± 5	90 ± 30
Liver	70 ± 10	7.5 ± 0.6	60 ± 10	80 ± 15

The amounts of released α-tocopherol from glycosides **2a–5a** after 2 h of incubation with 50 μL of supernatant from ileum, brain, liver, and kidney tissue supernatant are listed in Table 2. From the values of total released α-tocopherol in supernatants from the liver, kidneys, intestine, and brain were subtracted amounts of endogenic α-tocopherol: 0.154, 0.332, 0.307, and 0.385 nmol, respectively. The differences between total and endogenic content of α-tocopherol were shown as Δ parameter.

**TABLE 2:** Amounts of released α-tocopherol (nmol) after incubation of glycosides 2a–5a with 50 μL of supernatant fluid (n = 8)

	**2a** (nmol) ± SD		**3a** (nmol) ± SD		**4a** (nmol) ± SD		**5a** (nmol) ± SD		
					
Supernatant	α-toc.^a^	Δ_3_^b^	α-toc.^a^	Δ_1_^b^	α-toc.^a^	Δ_2_^b^	α-toc.^a^	Δ_4_^b^	endo (nmol)^c^
Liver	0.140 ± 0.5	−0.015 ± 0.0	0.921 ± 0.1	0.767 ± 0.23	3.217 ± 0.3	3.063 ± 0.45	0.156 ± 0.37	0.002 ± 0.00	0.154 ± 0.25
Kidney	0.923 ± 0.47	0.591 ± 0.17	1.072 ± 0.12	0.740 ± 0.2	1.034 ± 0.28	0.702 ± 0.4	0.327 ± 0.35	0.005 ± 0.00	0.332 ± 0.23
Intestine	2.276 ± 0.52	1.967 ± 0.2	2.618 ± 0.09	2.311 ± 0.25	2.502 ± 0.29	2.195 ± 0.42	0.323 ± 0.39	0.016 ± 0.00	0.307 ± 0.26
Brain	1.081 ± 0.49	0.696 ± 0.19	0.619 ± 0.11	0.234 ± 0.21	2.009 ± 0.31	1.624 ± 0.39	0.327 ± 0.37	−0.058 ± 0.00	0.385 ± 0.02

a Amounts of α-tocopherol released after incubation of the appropriate glycoside **2a–5a**.

b Amounts of released α-tocopherol diminished by the endogenic content.

c Endogenic content of α-tocopherol in 50 μL of supernatant fluid.

Due to the use of the marked excess of glycosides (1 mg), from each portion of glycosides **2a–5a** the samples of 1 mg were taken, and then extracted with hexane (3 × 1 mL). The combined hexane extracts were evaporated to dryness and dissolved in 0.5 mL of hexane. In the combined hexane extracts we did not observe by HPLC (at the detection limit of 1 × 10^−6^ mg/mL) the presence of free α-tocopherol.

The stability of *O*-glycosides of α-tocopherol **2a–5a** in the solution of citric buffer with an addition of BHT and ascorbic acid as antioxidants was also examined.

The samples of **2a–5a** (1 mg) were incubated according to Zwierz et al. (1981) using the same volume of citric buffer instead of tissue supernatant. The action of buffer solution was interrupted after 2, 4, 8, 12, and 24 h, respectively, by extraction with hexane (3 × 1 mL). The amounts of α-tocopherol released from the glycosides **2a–5a**, due to hydrolysis in buffer, are presented in Table 3.

**TABLE 3:** Amounts of released α-tocopherol (nmol) after incubation of 2a–5a glycosides in citric buffer (n = 8)

	Glycoside (nmol)			
	
Time of incubation (h)	**2a** ± SD	**3a** ± SD	**4a** ± SD	**5a** ± SD
2	0.100 ± 0.22^a^	0.070 ± 0.09^a^	0.116 ± 0.04^a^	0.078 ± 0.07^a^
4	0.145 ± 0.43^a^	0.098 ± 0.14^a^	0.198 ± 0.04^a^	0.124 ± 0.09^a^
8	0.433 ± 0.325^a^	0.350 ± 0.1^a^	0.366 ± 0.09^a^	0.471 ± 0.11^a^
12	0.970 ± 0.09^a^	0.584 ± 0.15^a^	1.279 ± 0.08^a^	0.528 ± 0.15^a^
24	1.729 ± 0.47^a^	0.926 ± 0.28^a^	1.391 ± 0.08^a^	0.602 ± 0.2^a^

a Amount of α-tocopherol released as a result of incubation of the glycosides **2a–5a** in citric buffer.

The comparison of the amounts of α-tocopherol released from the glycosides **2a–5a** incubated in tissue supernatants (Table 2) and the amount of released *p*-nitrophenol from the glycosides **2b–5b** (Table 1) showed that *O*-glycosidic bonds in derivatives **2a–5a** are more stable toward hydrolysis as compared with the appropriate *p*-nitrophenyl glycosides **2b–5b**.

A similar effect was observed previously for galactoside **4a** in rat tissue supernatants (Witkowski et al. 2004). According to the literature, the 2,6-dimethylphenyl glycosides are more resistant to hydrolysis by exoglycosidases than the glycosides of *p*-substituted phenols (Hall et al. 1961; Nath and Rydon 1954).

**FIGURE 4:**
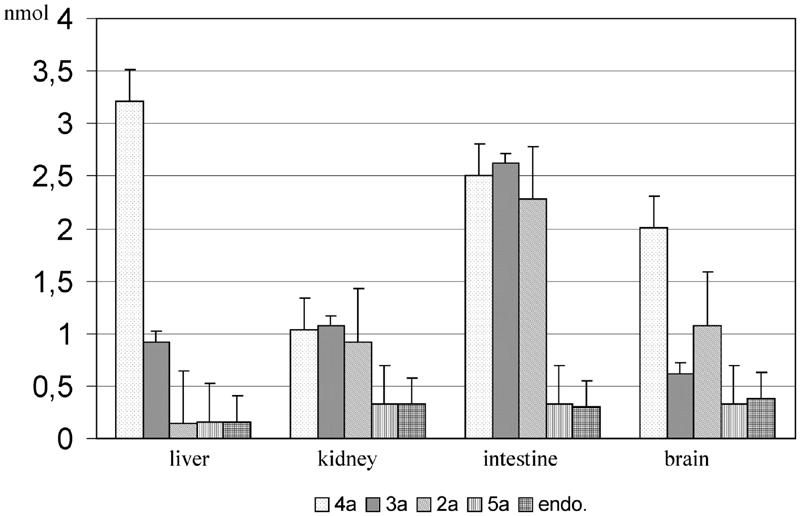
The comparison of released α-tocopherol after 2-h incubation of the glycosides 2a–5a with 50 μL of the specified supernatants and the endogenic content of α-tocopherol. The results are presented as the mean value ± standard deviation (n = 20).

A higher stability of glycosidic bonds in **2a–5a**, as compared to **2b–5b**, is a result of steric hindrance of the tocopheryl system, which hinders the effective contact of the active center of exoglycosidase with the substrate. However, the considerable amounts of free α-tocopherol (2–3 mmol) in the samples incubated with glycosides **3a** and **4a** in the respective supernatant of ileum and liver do not allow us to univocally conclude that the tested *O*-glycosides of α-tocopherol are completely passive toward endogenic glycosidases in supernatant fluids. It can be assumed that the presence of free α-tocopherol in the samples of **2a–5a** is the effect of cleavage of the glycosides under incubation conditions. The comparison of the amount of released α-tocopherol from glycosides **2a–5a** during a 2-h incubation with the endogenic content is presented in Figure 4.

Among tested glycosides of α-tocopherol, the considerable amounts of α-tocopherol were released during the incubation of the glycosides **2a–4a** in the supernatant of the ileum (1.967, 2.311, and 2.195 nmol, respectively) and galactoside **4a** in the supernatant of the liver (3.063 nmol).

Unexpectedly, the most persistent glycosides seem to be **2a** and **5a**. The 2-acetamidoglucoside **5a** does not practically undergo decomposition (Table 2). On the other hand, in case of *p*-nitrophenyl *O*-glycosides of **2b–5b**, it can be pointed out that the amount of released *p*-nitrophenol during incubation (Table 1) suggests that 2-acetamidoglucoside **5b** undergoes enzymatic hydrolysis easiest.

In case of tested supernatant fluids, the relative activity of the enzymes, determined on the basis of the amount of released *p*-nitrophenol, decreases as follows: N-acetyl-β-hexosaminidase > β-glucosidase > β-galactosidase > α-mannosidase. However, the described order does not occur in the case of α-tocopheryl glycosides **2a–5a**.

It can be suggested that α-tocopheryl glycosides undergo nonenzymatic hydrolysis due to environmental acidity (from citric buffer). Therefore, the stability of derivatives **2a–5a** in citric buffer was examined.

The incubation was conducted in the mixture of identical composition as applied in the examination in supernatants, using 50 μL of citric buffer instead of the same amount of supernatant. The hydrolysis was stopped after 2, 4, 8, 12, and 24 h, and the amount of released α-tocopherol was determined by HPLC (Table 3).

All the tested compounds underwent nonenzymatic hydrolysis and the amount of released α-tocopherol varied from 0.07 to 0.166 nmol after 2 h of incubation.

The values were considerably lower than those obtained after incubation with tissue supernatants (Table 2). This suggests that α-tocopherol from *O*-glycosides is released in the way of enzymatic and nonenzymatic hydrolysis.

**FIGURE 5:**
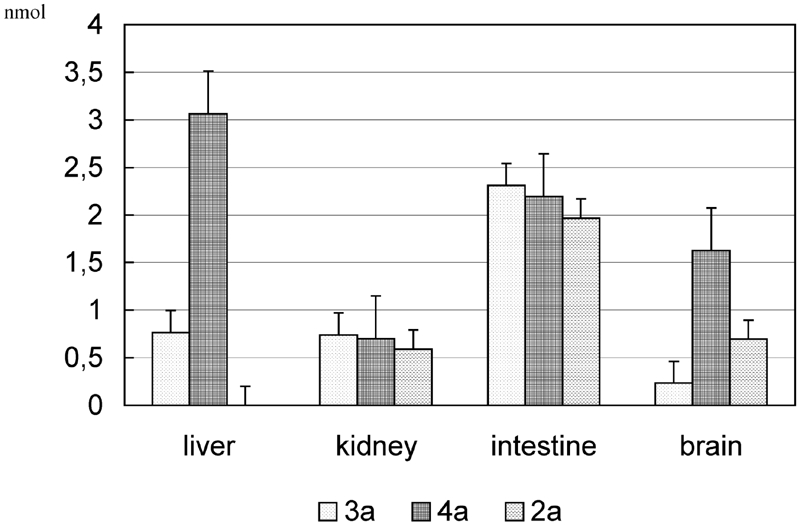
Amounts of α-tocopherol released from glycosides 2a–4a as a result of 2-h hydrolysis, diminished by the endogenic content α-tocopherol. The results (n = 10) are presented as the mean value ± standard deviation.

The amounts released from supernatants α-tocopherol after a 2-h incubation diminished by the endogenic content and the value obtained after hydrolysis in citric acid are presented in Figure 5.

The above results point out that the tested glycosides **2a–4a** most efficiently undergo enzymatic hydrolysis in the ileum supernatant.

According to the literature, the more axial hydroxyl groups there are in sugar residue, the higher the rate of nonenzymatic hydrolysis of *O*-glycosides (Helligso and Bols 2003).

**FIGURE 6:**
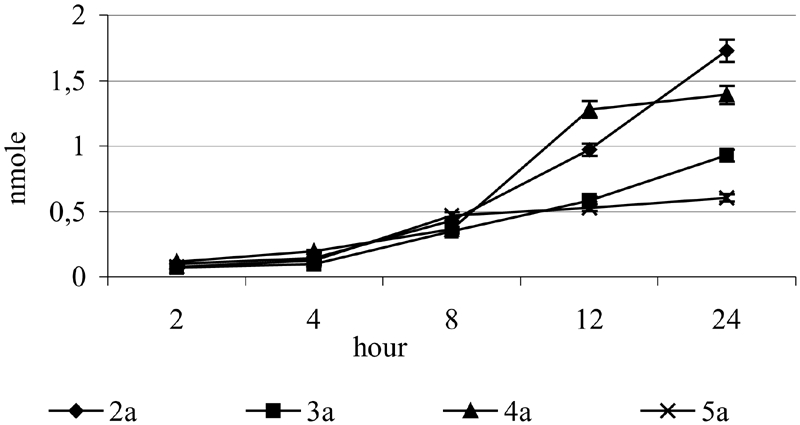
Amount of α-tocopherol released as a result of hydrolysis of the glycoside 2a–5a in buffer. The results (n = 10) were presented as the mean value ± standard deviation.

Thus, the galactoside **4b** is easily cleavable both enzymatically and nonenzymatically. Unexpectedly, 2-acetamidoglucosides of α-tocopherol **5a** appeared to be the most stable among the tested glycosides. However, prolonged time of hydrolysis caused a slow hydrolysis of 2-acetamideglycoside (Fig. 6).

The obtained results show that the examined glycosidic derivatives **2a–5b**, especially in the tissue of ileum, can undergo a prolonged cleavage with the release of free α-tocopherol.

## CONCLUSION

The stability of glycosides of α-tocopherol **2a–5a** in the presence of exoglycosidases that are present in supernatants of rat tissues (liver, ileum, kidney, and brain) was evaluated. The results show that *O*-glycosides of tocopherol **2a–5a** are more resistant to enzymatic hydrolysis as compared with the respective *p*-nitrophenyl *O*-glycosides **2b–5b**.

Among the tested derivatives, the galactoside **4a** easily undergoes hydrolysis in supernatant fluid from ileum. The slow decomposition of α-tocopherol *O*-glycosides in tissues can be beneficial to obtain the vitamin E derivatives with prolonged action in the organism.
